# The association between *MTHFR* 677C>T genotype and folate status and genomic and gene-specific DNA methylation in the colon of individuals without colorectal neoplasia^1^,^2^,^3^,^4^

**DOI:** 10.3945/ajcn.113.061432

**Published:** 2013-10-09

**Authors:** Joanna Hanks, Iyeman Ayed, Neil Kukreja, Chris Rogers, Jessica Harris, Alina Gheorghiu, Chee Ling Liu, Peter Emery, Maria Pufulete

**Affiliations:** 1 From the Diabetes and Nutritional Sciences Division, King's College London, London, United Kingdom (J Hanks, IA, NK, AG, CLL, PE, and MP), and the Clinical Trials and Evaluation Unit, School of Clinical Sciences, University of Bristol, Bristol, United Kingdom (CR, J Harris, and MP).

## Abstract

**Background:** Decreased genomic and increased gene-specific DNA methylation predispose to colorectal cancer. Dietary folate intake and the methylenetetrahydrofolate reductase polymorphism (*MTHFR* 677C>T) may influence risk by modifying DNA methylation.

**Objective:** We investigated the associations between *MTHFR* 677C>T genotype, folate status, and DNA methylation in the colon.

**Design:** We conducted a cross-sectional study of 336 men and women (age 19–92 y) in the United Kingdom without colorectal neoplasia. We obtained blood samples for measurement of serum and red blood cell folate, plasma homocysteine, and *MTHFR* 677C>T genotype and colonic tissue biopsies for measurement of colonic tissue folate and DNA methylation (genomic- and gene-specific, estrogen receptor 1, *ESR1*; myoblast determination protein 1, *MYOD1*; insulin-like growth factor II, *IGF2*; tumor suppressor candidate 33, *N33*; adenomatous polyposis coli, *APC*; mut-L homolog 1, *MLH1*; and O^6^-methylguanine-DNA methyltransferase, *MGMT*) by liquid chromatography/electrospray ionization mass spectrometry and pyrosequencing, respectively.

**Results:** Of the 336 subjects recruited, 185 (55%) carried the CC, 119 (35%) the CT, and 32 (10%) the TT alleles. No significant differences in systemic markers of folate status and colonic tissue folate between genotypes were found. The *MTHFR* TT genotype was not associated with genomic or gene-specific DNA methylation. Biomarkers of folate status were not associated with genomic DNA methylation. Relations between biomarkers of folate status and gene-specific methylation were inconsistent. However, low serum folate was associated with high *MGMT* methylation (*P* = 0.001).

**Conclusion:** *MTHFR* 677C>T genotype and folate status were generally not associated with DNA methylation in the colon of a folate-replete population without neoplasia. This trial was registered at clinicaltrials.gov as ISRCTN43577261.

## INTRODUCTION

There is conflicting evidence regarding the role of folate in colorectal cancer. Meta-analyses of cohort studies generally point to a negative association between folate intake and risk (1–3). More recently, 2 US cohorts reporting postfortification dietary folate intakes showed significant negative associations (4, 5), whereas another US cohort showed no effect (6). Results from randomized controlled trials investigating whether folic acid supplementation decreases the risk of colorectal adenoma (precursor of colorectal cancer) are largely negative (7–10), although the follow up of these trials has been probably too short to result in changes in risk of colorectal cancer, which develops over decades.

Folate provides one-carbon units for DNA methylation and DNA synthesis, and disruption in both of these processes is the hallmark of neoplasia. Modifications to DNA methylation occur on a genomic- and gene-specific level in colorectal neoplasia: a decrease in genomic DNA methylation (hypomethylation) (11) is accompanied by an increase in methylation of CpG islands in the promoter regions of many tumor-suppressor genes (hypermethylation) (12, 13).

It is uncertain whether folate status influences DNA methylation in the colon. Some reports have suggested associations between serum and red blood cell folate and genomic DNA methylation in people with and without neoplasia (14, 15), whereas others found none (16). One study found a positive association between red blood cell folate and hypermethylation of estrogen receptor 1 (*ESR1*)^5^ and secreted frizzled-related protein 1 (*SFRP1*) genes in the colon of subjects with adenoma (17), whereas another found no associations between markers of folate status and methylation in *ESR1* and mut-L homolog 1 (*MLH1*) (18). Folic acid intervention trials in individuals with adenoma have reported equally mixed results, with some suggesting that supplementation can reverse genomic DNA hypomethylation in the colon (19, 20) and others showing no effect on gene-specific methylation (17, 21).

The common 677C>T polymorphism in the gene encoding the key enzyme methylenetetrahydrofolate reductase (*MTHFR*) provides an additional factor that could modify associations between folate status and DNA methylation in the colon. Although associated with a lower risk of colorectal cancer (22), the exact interaction of this polymorphism with DNA methylation in the colon is not known, although interactions with folate status and genomic DNA methylation in leukocytes have been reported (23, 24).

The current study was designed to assess the association between the *MTHFR* 677C>T polymorphism, markers of folate status, and genomic- and gene-specific DNA methylation in the colon of subjects without colorectal neoplasia. We investigated gene-specific DNA methylation in 7 tumor suppressor genes: *ESR1*, myoblast determination protein 1 (*MYOD1*), insulin-like growth factor II (*IGF2*), tumor suppressor candidate 33 (*N33*), adenomatous polyposis coli (*APC*), *MLH1*, and O^6^-methylguanine-DNA methyltransferase (*MGMT*). These genes were selected because they all show increased methylation and silencing in colorectal tumors (25), and some also show age-related increases in methylation in the normal colonic mucosa of individuals with and without colorectal neoplasia (18, 26–30).

## SUBJECTS AND METHODS

### Study population

Subjects were patients (>18 y of age with no upper age limit) who were referred for a clinically indicated colonoscopy at the Endoscopy Departments at King's College Hospital and Guy's and St Thomas’ Hospital NHS Foundation Trusts between December 2006 and February 2009. Subjects were eligible for inclusion if they had a normal colonoscopy (to the ileo-caecal valve) with no macroscopic evidence of colorectal tumors (polyps or cancer) or inflammation. Subjects were excluded if they had any of the following: tumors at current colonoscopy; a strong family history of colorectal cancer as defined in the British Society of Gastroenterology guidelines (one first-degree relative with colorectal cancer younger than 45 y; 2 first-degree relatives with colorectal cancer; a family history of endometrial and colorectal cancer, where one colorectal cancer occurs in a relative younger than 50 y); a history of familial polyp syndromes; history of colorectal tumors; inflammatory bowel disease; a current or history of gluten-sensitive enteropathy; documented renal or liver disease; pregnancy; epilepsy; alcoholism; pernicious anemia; and use of antifolate medication. Ethical approval was obtained from King's College Hospital NHS Trust (REC number: 06Q0703/29).

Patients were approached at the preassessment stage before admission to the Endoscopy Department. Initially, medical notes were screened to exclude those who clearly did not meet the inclusion criteria; those who were deemed suitable were approached, informed about the study, and asked for consent. On the day of colonoscopy, those who provided written consent were asked to complete a health screening questionnaire that collected basic demographic information and information on smoking habits, alcohol intake, current medication, and supplement use. Subjects were defined as supplement users if they reported taking any nutritional supplements [vitamins (including folic acid), minerals, amino acids, and fatty acids] at any time in the previous 3 mo. Subjects were defined as folic acid supplement users if they reported taking folic acid supplements or multivitamin preparations containing folic acid at any time in the previous 3 mo. Subjects were also weighed and their heights were measured and BMI calculated as weight/height^2^. All patients had prepared for colonoscopy by consuming an orally administered colonic lavage solution (KleanPrep; Norgine).

A fasting blood sample was collected before colonoscopy by using the evacuated tube technique (red top containing no anticoagulant for serum folate and vitamin B-12 and liver function tests and purple top containing EDTA for full blood count, red blood cell folate, plasma homocysteine, and DNA extraction). The EDTA-treated samples for plasma homocysteine were chilled on ice. Blood samples were spun within 2 h of collection; serum and plasma were stored at −80°C until assayed. At the end of colonoscopy, 6 mucosal biopsies were removed from the rectum (∼12 cm from the anal verge) and immediately snap-frozen in liquid nitrogen. Of these, only 4 biopsies were available for use in the current study. After colonoscopy, subjects were given a food-frequency questionnaire (31) designed to assess dietary folate intake to take home and complete; they were asked to return the completed food-frequency questionnaire in the stamped addressed envelope provided.

### Sample size and primary endpoints

The primary endpoints were differences in genomic- and gene-specific DNA methylation between the *MTHFR* 677C>T genotypes (CC, CT, and TT). Because there are no data on differences in gene-specific methylation between genotypes, sample size was calculated by using data from a previous observational study in which leukocyte DNA methylation was measured in healthy volunteers (24). On the basis of differences in genomic DNA methylation between CC and TT individuals in that study, a comparison between 32 TT individuals and 128 CC individuals would be adequate to detect a 50% difference (0.5 SD, 30 ng mCyt/μg DNA) in genomic DNA methylation between CC and TT individuals (5% significance level, 2 tailed, and 80% power; specifying the ratio of CC to TT as 4).

### Biochemical analyses

Markers of folate status (serum and red blood cell folate and plasma homocysteine), serum vitamin B-12, and liver function variables were all measured in the Departments of Clinical Biochemistry and Haematology at King's College Hospital NHS Trust, London. Liver function was determined by using the Advia 2400 Chemistry System (Siemens Health Care Diagnostics). The hematocrit (required to calculate red blood cell folate) was measured in fresh whole blood within 24 h of collection by using an ADVIA 2120 Hematology System (Siemens Health Care Diagnostics). Serum folate, vitamin B-12, red blood cell folate, and plasma homocysteine were analyzed by competitive protein binding enzyme immunoassay with direct chemiluminescent technology on an Advia Centaur XP Immunoassay System (Siemens Health Care Diagnostics). For both folate and homocysteine assays, the within-run CV was <2.2% and the between-run CV was <5.2%.

### Genotyping for the *MTHFR* 677C>T mutation

DNA was extracted from whole blood by using the GenElute Blood Genomic DNA Kit (Sigma-Aldrich). The DNA was quantified and assessed for purity by using the Nano Drop ND-1000 ultraviolet spectrophotometer (Thermo Scientific) and run on agarose gels to determine size (> 20 kb for all samples). The presence of the *MTHFR* 677C>T mutation was determined by DNA amplification with appropriate forward and reverse primers followed by *Hinf I* digestion of polymerase chain reaction (PCR) product as previously described (32).

### Tissue folate analysis

Folate concentrations in colorectal biopsies were measured by standard microbiological assay with the use of *Lactobacillus rhamnosus* (ATCC 7469) (33). Briefly, tissue biopsies (5–64 mg) were weighed and homogenized (by crushing and shearing the sample in a standard Eppendorf tube with a polytetrafluoroethylene pestle until the sample was barely visible) in 20 volumes of folate extraction buffer [5 mmol β-mercaptoethanol/L and 0.1 mol sodium ascorbate/L in 0.1 mol *bis*(2-hydroxyethyl)imino-tris(hydroxymethyl) methane/L, pH 7.85] and immersed in boiling water for 20 min. The homogenate was cooled in an ice bath and centrifuged at 4°C, 2000 rpm, for 10 min. The supernatant fluid (1 mL) was transferred to sterile Eppendorf tubes and deconjugated with 1 mL chicken pancreas solution (5 mg/mL) and 3 mL 0.1 mol sodium phosphate buffer/L for 16 to 18 h at 37°C to convert folylpolyglutamates to corresponding diglutamate derivatives. Deconjugated folate samples were incubated with *L. rhamnosus* on 96-well plates for 42 h at 37°C. Plates were read on a plate reader at 620 nm. Folate concentrations in the biopsies were calculated from a standard curve of serial dilutions of a folic acid standard. Folate extracted from pooled colorectal tissue samples served as the quality control (QC). For this assay, the within-run CV (30 QC samples) was 3.2% and the between-run CV (QC sample assayed over 4 d) was 6.8%.

### DNA extraction from tissue biopsies

Genomic DNA was extracted (simultaneously with RNA) from the tissue biopsies by using the AllPrep DNA/RNA Mini Kit (Qiagen). Before extraction, frozen samples were disrupted with a micropestle on dry ice followed by homogenization with the QIAshredder (Qiagen). DNA was quantitated and assessed for purity and size as described in the section on genotyping.

### Genomic DNA methylation

Genomic DNA methylation was determined by liquid chromatography/electrospray ionization mass spectrometry (LC/ESI-MS); 1–5.6 μg DNA was denatured by heating to 100°C for 5 min and then immediately chilled on ice followed by addition of 0.45 μg [^13^C,^15^N_2_]2′-deoxycytidine (TRC Toronto Research Chemicals Inc) (the internal standard) and 1/10 volume of 0.1 mol ammonium acetate/L. The mixture was then digested with 4 U S1 nuclease (Sigma-Aldrich) for 2 h at 45°C, 0.004 U snake venom phosphodiesterase (GE Health Care), 1/10 volume of 1 mol ammonium bicarbonate/L for 2 h at 37°C, and 1 U shrimp alkaline phosphatase (GE Health Care) for 1 h at 37°C.

Digested DNA (40 μL) was chromatographed on a Phenomenex Synergi Polar RP column (250 × 2 mm, 4-μm particle size, 80-Å pore size) by using an isocratic mobile phase consisting of 10 mmol/L ammonium formate solution + 0.1% formic acid:methanol (95:5) pumped at a flow rate of 0.2 mL/min. The column temperature was 30°C, and the sample rack temperature was 4°C. The eluent was diverted from the mass spectrometer to waste from 0 to 3 min. The total run time was 10 min.

The chromatography column was coupled to a Micromass Quattro Ultima tandem mass spectrometer (Micromass Quattro Ultima Altrincham) operating in ESI-positive mode by using the following settings: source block temperature of 120°C, desolvation temperature of 450°C, cone gas (N_2_) flow of ∼100 L/h, desolvation gas (N_2_) flow of ∼650 L/h, collision gas (Ar) pressure of ∼2.7 × 10^−3^ mbar, capillary voltage of 2.5 KV, cone voltage of 15 V. The instrument was operated at unit mass resolution (LM & HM, 1& 2 resolution set to 13), collision energy voltage of 12 V, and multiplier voltage of 650V.

2′-Deoxycytidine and 5-methyl-2′-deoxycytidine (Sigma-Aldrich) were used as external standards. Quantification was carried out in multiple reaction monitoring mode by monitoring the precursor/product ion pair of *m/z* 228/112 for 2′-deoxycytidine and *m/z* 242/126 for 5-methyl-2′-deoxycytidine; the dwell time was 500s for each pair. The chromatographic peaks for 2′-deoxycytidine and 5-methyl-2′-deoxycytidine eluted at 5.0 and 6.5 min, respectively. Chromatograms were analyzed by using the Quanlynx v 4.1 software package.

The cytosine and 5-methylcytosine contents in DNA were calculated from calibration curves, where the area ratios of 2′-deoxycytidine or 5-methyl-2′-deoxycytidine to internal standard (labeled deoxycytidine) were plotted against known concentrations of 2′-deoxycytidine or 5-methyl-2′-deoxycytidine. The genomic DNA methylation status was expressed as the relative amount of 5-methyl-2′-deoxycytidine compared with total deoxycytidine residues (ie, % methylcytosine). The within- and between-day CVs (determined from QC samples assayed on the same day and on 12 different days) were 3.9% and 10.0%, respectively.

### Gene-specific DNA methylation

Bisulfite modification of DNA (500 ng per sample) was carried out by using the EZ-96 DNA Methylation-Gold Kit (Zymo Research Corp) according to the manufacturer's protocol. Bisulfite-modified DNA was stored at −20°C until used.

Promoter sequences were obtained from the ENSEMBL human genome database (http://www.ensembl.org/Homo_sapiens/Info/Index). Primers were designed by using the PSQ Assay Design Software (Qiagen) to amplify a maximum length of 200 bp of the selected 1000-bp sequence upstream of the promoter of each gene. Forward and reverse (biotinlyated) primer sets (5′-3′) for each gene were as follows: *ESR1* (GAGGTGTATTTGGATAGTAGTAAGTT; CTATTAAATAAAAAAAAACCCCCCAA);* MYOD1* (AGGGGATAGAGGAGTATTGAAAG; CCCCCAAACTAACCAACCA); *IGF2* (GGGGGAGTTGAGGTAGATGAA; CCCCCCCCTCCTAACCCAC);* N33* (GGTAGAGGAAGTAGAGGAGGAGAT; AAAACCAAAAAACCACTCCTCTC); *MLH1* (AGTTTTTTTTTTAGGAGTGAAGG; TAAAACCCTATACCTAATCTATC); *MGMT* (GGGGGAGTTGAGGTAGATGAA; GGATATGTTGGGATAGTT); and* APC* (AGGGTGAGATATGGAGAGAAC; ACCCTCAATTCTCCAA).

The PCR mix (50 μL volume) included the following: 50 ng bisulfate-converted DNA, 10 pmol forward and reverse primer, 10× PCR buffer (containing 1.5 mmol MgCl_2_/L), and an additional 0–1.5 mmol MgCl_2_/L, 200 μmol/L of each dNTP, 1.25 U HotStarTaq DNA polymerase (Qiagen), and nuclease-free water to make up to final volume. The final concentration of MgCl_2_ ranged from 1.5 to 3.0 mmol/L. The cycling conditions were as follows: 95°C for 15 min; 50 cycles of 95°C for 20 s, 54–60°C for 20 s, and 72°C for 20 s; 72°C for 5 min; and 4°C hold. Gel electrophoresis was carried out on all PCR products by using 10 μL PCR product, which was loaded onto a 1.5% (wt:vol) agarose gel. The gels where then visualized by using ultraviolet transilluminator (Alpha Imager; Alpha Innotech Corporation).

Pyrosequencing was carried out by using the PSQHS 96 system (Qiagen). Sample preparation was carried out by using the PyroMark Vacuum Prep Workstation (Qiagen) according to the manufacturer's protocol: 10 μL PCR product (biotinylated strand only) was immobilized to 2 μL Streptavidin Sepharose HP beads (GE Health Care) by using the Vacuum Prep Workstation; the beads were released onto a PSQ HS 96 plate containing 10 pmol sequencing primer (*ESR1*, GAGGGGTGTTTAGAGTTT; MYOD1, GAAAGTTAGTTTAGAGGTGA; *IGF2*, GAGGTAGATGAAGAGGA; N33, AGGAGGAGATTGATAGAGTA; *MLH1*, GTAGTATTGTGTTTAGTTT; *MGMT*, GATTTGGTGAGTGTTTGGGT; *APC*, GGAGAGAAGAGAGTTATAG), and the plate was heated to 80°C for 2 min to anneal the primers. Sequencing was carried out on the PSQHS 96 system according to standard procedures. The Pyro Q-CpG (Qiagen) software was used to quantify CpG methylation. The degree of methylation at each CpG site was calculated as a percentage by using the allele quantification functionality of the PSQHS 96 software and is expressed as a percentage [methylation (%) = peak height methylated/(peak height methylated – peak height nonmethylated) × 100]. For each gene, we calculated the average methylation (%) across all CpG dinucleotides in the sequence studied.

Universal methylated and unmethylated control DNA (Zymo Research Corp) were used as positive and negative controls. A pooled colorectal tissue DNA sample was bisulfite modified and served as the QC for the entire set of genes. Within-run and between-run CVs for all assays were 3% and 7%, respectively.

All laboratory personnel who carried out the blood and tissue folate and plasma homocysteine analysis and the genomic- and gene-specific methylation analysis were blinded to the *MTHFR* 677C>T genotype status of participants.

### Statistical analysis

Data were analyzed by using STATA 11 for Windows (StataCorpLP). Differences in categorical variables (sex, ethnic group, smoking, and supplement use) between genotypes were compared by using chi-square tests. Differences in single continuous variables between genotypes were compared by using ANOVA (age, BMI, dietary folate intake, and genomic DNA methylation) or Kruskal-Wallis tests, which were used for variables that did not have a normal distribution or for which there was a violation of the equality of variance assumption (alcohol intake, serum, red blood cell and colonic tissue folate, serum vitamin B-12, plasma homocysteine, and gene-specific methylation). The relation between demographic variables (age, sex, ethnic group, BMI, smoking, supplement use, and alcohol intake), biomarkers of folate status (serum, red blood cell and colonic tissue folate, and plasma homocysteine), *MTHFR* 677C>T genotype, and genomic- and gene-specific DNA methylation was investigated by using Spearman's rank correlation coefficients. The association between plasma homocysteine and demographic variables, biomarkers of folate status, serum vitamin B-12, and *MTHFR* 677C>T genotype was assessed by using multiple linear regression. Plasma homocysteine was skewed, and no transformation improved the model fit; therefore, we performed the regression analysis after removing 4 outliers. Removal of these outliers did not change the conclusions.

Genomic- and gene-specific DNA methylation values are expressed as a proportion and are therefore bounded between 0 and 1. We assessed the association between genomic- and gene-specific methylation and biomarkers of folate status and *MTHFR* 677C>T genotype by using generalized linear models with the binomial family distribution and logit link function, including the robust option to obtain robust SEs. The coefficient from the model gives the amount of change in the log odds of having high methylation for each unit increase in biomarkers of folate status. All models were adjusted for age (continuous), sex (male or female), ethnic group (white or nonwhite), supplement use (user or nonuser), and serum vitamin B-12 (continuous). For genomic DNA methylation, both unadjusted and adjusted models are presented. For gene-specific methylation, we presented only the adjusted models because the differences between adjusted and unadjusted models were marginal. For all models, we checked model fit visually using plots of residuals versus fitted values and versus each of the predictors in turn and normal QQ plots of standardized deviance residuals. We used Stata's linktest to check whether logit was the correct link function to use and assessed model fit by using the Hosmer and Lemeshow goodness-of-fit test and Pearson's correlation test. For all models, results of these diagnostic tests suggested good model fit (*P* > 0.05). We corrected for multiple comparisons (8 repeated analyses) by using the Bonferroni correction to reduce the chance of obtaining false-positive results (type I error). We therefore considered a *P* value of 0.006 to be significant (0.05/8). All statistical tests were 2 sided. We repeated the analysis without adding supplement use to the models to prevent possible overadjustment (because supplement use is clearly linked with the provision of B vitamins). The results were not materially altered (data not shown). For gene-specific methylation, we presented coefficients and 95% CIs in graphic form for ease of interpretation.

## RESULTS

Of the 3112 patients screened, 295 did not consent to participate and 2466 did not meet the inclusion criteria, leaving a total of 351 eligible subjects. Of these, 4 had no blood biochemistry results and 11 had abnormal liver function tests; this left a total of 336 participants for analysis (96% of those eligible). Of these, 185 (55%) were homozygous for the wild-type allele (CC) of the *MTHFR* 677C>T genotype, 119 (35%) carried one mutant allele (CT) and 32 (10%) were homozygous for the mutant allele (TT). Subject characteristics are shown in **Table 1**. There were no significant differences in age, BMI, folate or alcohol intakes, or the distribution of sex, smoking, and supplement use between genotypes. The distribution of white and black subjects differed between genotypes (white: 50% CC, 39% CT, and 11% TT; black: 72% CC, 23% CT, and 4% TT). Only 60 subjects (18%) returned the food-frequency questionnaire, hence dietary folate intakes were only available for these individuals.

**TABLE 1:** Subject characteristics by *MTHFR* 677C>T genotype

		CC (*n* = 185)	CT (*n* = 119)	TT (*n* = 32)	*P* value*^1^*
Age (y)		57 (55, 59)*^2^*	57 (54, 60)	54 (48, 60)	0.58
Sex [*n* (%)]					0.36
Male		67 (36)	51 (43)	10 (31)	
Female		118 (64)	68 (57)	22 (69)	
Ethnic group [*n* (%)]					0.01*^3^*
White		114 (62)	87 (73)	25 (78)	
Black		50 (27)	16 (13)	3 (9)	
Asian		11 (6)	5 (4)	2 (6)	
Other		5 (3)	5 (4)	1 (3)	
Not recorded		5 (3)	6 (5)	1 (3)	
BMI (kg/m^2^)		26.9 (25.9, 28.0)	26.7 (25.7, 27.7)	27.6 (25.2, 29.9)	0.80
Smoking [*n* (%)]					
* n*		172	112	30	
Nonsmoker		132 (77)	87 (78)	20 (67)	0.44
Smoker		40 (23)	25 (22)	10 (33)	
Supplement use [*n* (%)]					0.80
* n*		168	111	30	
Nonuser user		114 (68)	78 (70)	22 (73)	
User		54 (32)	33 (30)	8 (27)	
Folate-containing supplement use [*n* (%)]					
* n*		168	111	30	
Nonuser		145 (86)	94 (85)	26 (87)	
User		23 (14)	17 (15)	4 (13)	
Dietary folate intake (μg/d)*^4^*		327 (287, 366)	376 (316, 436)	370 (284, 455)	
Total folate intake (diet + supplements) (μg/d)		345 (297, 393)	415 (335, 495)	392 (322, 462)	0.21
Alcohol intake (g/d)		1 (0–78)*^5^*	1 (0–103)	1 (0–64)	0.59

1 Differences between genotypes were tested by using chi-square tests (sex, smoking, and supplement use), ANOVA (age, BMI, and folate intake), and the Kruskal-Wallis test (alcohol intake).

2 Mean; 95% CI in parentheses (all such values).

3 The chi-square test was for white compared with black because of the limited number of subjects in the other groups.

4 = 33, 18, and 9 for the CC, CT, and TT groups, respectively.

5 Median; range in parentheses (all such values).

The biomarkers of folate and vitamin B-12 status by *MTHFR* 677C>T genotype are shown in **Table 2**. There were no significant differences in serum, red cell and tissue folate, serum vitamin B-12 or plasma homocysteine between genotypes. Serum and red cell folate were positively correlated (ρ = 0.525, *P* < 0.001); both were negatively correlated with plasma homocysteine (ρ = −0.309 and ρ = −0.261, *P* < 0.001, respectively). Tissue folate was positively correlated with serum and red cell folate (ρ = 0.464 and ρ = 0.292, *P* < 0.001) and negatively correlated with plasma homocysteine (ρ = −0.140, *P* = 0.01). There were no significant correlations between dietary folate intake and any of the biomarkers of folate status in the subset of subjects (*n* = 60) who completed the food-frequency questionnaire (*P* > 0.05). However, the use of supplements containing folic acid were significantly correlated with serum folate (ρ = 0.200, *P* < 0.001), tissue folate (ρ = 0.121, *P* = 0.03) and plasma homocysteine (ρ = −0.205, *P* < 0.001), and weakly with red blood cell folate (ρ = 0.100, *P* = 0.08).

**TABLE 2:** Biomarkers of folate and vitamin B-12 status by *MTHFR* 677C>T genotype*^1^*

	CC (*n* = 185)	CT (*n* = 119)	TT (*n* = 32)	*P* value*^2^*
Serum folate (nmol/L)	26.1 (7.5–54.4)	24.5 (2.5–54.4)	25.4 (8.6–54.4)	0.43
Red blood cell folate (nmol/L)	977 (372–2234)	927 (406–1752)	969 (605–2982)	0.44
Colonic tissue folate (nmol/g tissue)	0.89 (0.14–7.53)	0.88 (0.10–7.34)	0.90 (0.10–5.41)	0.91
Serum vitamin B-12 (pmol/L)	314 (89–1476)	315 (137–1117)	285 (123–662)	0.51
Plasma homocysteine (μmol/L)	15.6 (1.2–37.0)	16.2 (2.6–41.9)	17.8 (5.9–59.4)	0.27

1 All values are medians; ranges in parentheses.

2 Kruskal-Wallis tests were used to test for differences between genoytpes.

Multiple linear regression analysis with plasma homocysteine as the dependent variable showed associations with age (*P* < 0.001), sex (with higher homocysteine concentration in men, *P* < 0.001), serum vitamin B-12 (*P* = 0.001) and serum folate (*P* = 0.002) but no association with MTHFR 677C>T genotype (*P* > 0.05).

### Genomic DNA methylation

**FIGURE 1.**
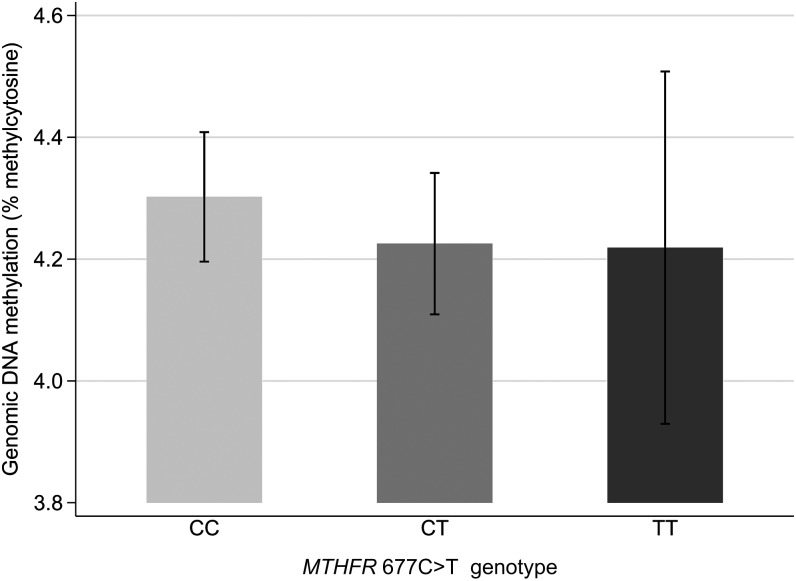
Genomic DNA methylation by *MTHFR* 677C>T genotype. Bars represent means and 95% CIs for unadjusted values (CC, *n* = 185; CT, *n* = 119; TT, *n* = 32; *P* = 0.60). *P* = 0.60 for comparison between genotypes (ANOVA).

The average methylcytosine content of colonic DNA was 4.2% with a range of 1.5–6.8%. There was no influence of *MTHFR* 677C>T genotype on genomic DNA methylation (*P* = 0.60, **Figure 1**). There was no correlation between genomic DNA methylation and serum, red blood cell or tissue folate, serum vitamin B-12 or plasma homocysteine (all *P* values >0.05, data not shown). The coefficients and 95% CIs (adjusted and unadjusted) from generalized linear models for the association between genomic DNA methylation and biomarkers of folate status and *MTHFR* 677C>T genotype are shown in **Table 3**. In the adjusted model, there were no significant associations between any of the folate biomarkers or *MTHFR* 677C>T genotype and genomic DNA methylation. In the unadjusted model, a significant negative association was found between genomic DNA methylation and red blood cell folate (*P* = 0.04), but this disappeared after adjustment (*P* = 0.07).

**TABLE 3:** Association between genomic DNA methylation in colonic mucosa, *MTHFR* 677C>T genotype, and biomarkers of folate status*^1^*

	Unadjusted coefficient (95% CI)	*P**^2^*	Adjusted coefficient (95% CI)*^3^*	*P**^2^*
*MTHFR* 677C>T				
CC (reference)				
CT	−0.0188 (−0.0570, 0.0194)	0.33	−0.0237 (−0.0639, 0.0165)*^4^*	0.25
TT	−0.0204 (−0.0929, 0.0520)	0.58	−0.0241 (−0.1024, 0.0541)*^4^*	0.55
Serum folate (nmol/L)	0.0003 (−0.0010, 0.0015)	0.70	0.0002 (−0.0012, 0.0169)	0.75
Red blood cell folate (nmol/L)	−0.0001 (−0.0001, −0.0000)	0.04	−0.0001 (−0.0001, 0.0000)	0.07
Colonic tissue folate (nmol/g tissue)	0.0091 (−0.0175, 0.0357)	0.50	0.0198 (−0.0007, 0.0403)	0.06
Plasma homocysteine (μmol/L)	0.0008 (−0.0017, 0.0034)	0.54	0.0009 (−0.0020, 0.0039)	0.62

1 Generalized linear model with a logit link (the natural log of the odds: log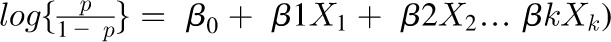). For the continuous predictor variables (biomarkers of folate status), the coefficient gives the amount of change in the log odds of having high methylation for each unit increase in the biomarker (ie, a positive coefficient suggests an increase in the odds, whereas a negative coefficient suggests a decrease in the odds of having high methylation with increasing biomarker concentration). For the categorical predictor variable (*MTHFR* 677C>T), the coefficient gives the log odds of having high methylation in the CT or TT genotype compared with the CC genotype (reference group), ie, a negative coefficient suggests a decrease in the odds of having high methylation in individuals carrying the CT or TT genotype compared with individuals carrying the CC genotype.

2 Threshold for significance using Bonferroni correction for 8 repeated tests is *P* = 0.006.

3 Adjusted for age, sex (male or female), ethnicity (white or nonwhite), supplement use (user or nonuser), serum vitamin B-12, and *MTHFR* 677C>T genotype.

4 Adjusted for age, sex (male or female), ethnicity (white or nonwhite), supplement use (user or nonuser), and serum vitamin B-12.

### Gene-specific methylation

The median (range) methylation across all CpG sites analyzed for *ESR1*, *MYOD1*, *IGF2*, and *N33* were as follows: *ESR1*, 13% (1–40%); *MYOD1*, 10% (2–28%); *IGF2*, 8% (1–33%); and *N33*, 14% (3–67%). For *APC*, *MLH1*, and *MGMT*, the medians (ranges) were as follows: *APC*, 2% (0–7%); *MLH1*, 2% (0–31%); and *MGMT*, 2% (0–5%). *MLH1* had a small proportion of subjects (15%) with methylation values >10% (11–31%), but most (85%) had values between 0% and 10%.

*ESR1*, *N33*, and *MYOD1* methylation were positively associated with age (ρ = 0.373, ρ = 0.29, and ρ = 0.46, respectively; *P* < 0.001) and with each other (*ESR1* and *MYOD1*, ρ = 0.408; *ER* and *N33*, ρ = 0.247; *N33* and *MYOD1*, ρ = 0.233, *P* < 0.001). *IGF2*, *APC*, *MLH1*, and *MGMT* methylation were not associated with age (*P* > 0.05). *APC* methylation was positively associated with *IGF2* methylation (ρ = 0.117, *P* = 0.03) and *MGMT* methylation (ρ = 0.135, *P* = 0.01). *MLH1* methylation was positively associated with *ESR1* methylation (ρ = 0.110, *P* = 0.04). No association was found between genomic- and gene-specific DNA methylation (*P* > 0.05).

Of the other demographic variables assessed, sex was associated with *IGF2* methylation (16% higher in men than in women; *P* = 0.02), ethnicity (white compared with nonwhite) was associated with *N33* methylation (5% higher in nonwhites; *P* = 0.04), and supplement use was associated with *APC* methylation (17% lower in users; *P* = 0.03). No other significant associations were noted. All subsequent statistical analyses were adjusted for age, sex, ethnic group, and supplement use.

**FIGURE 2.**
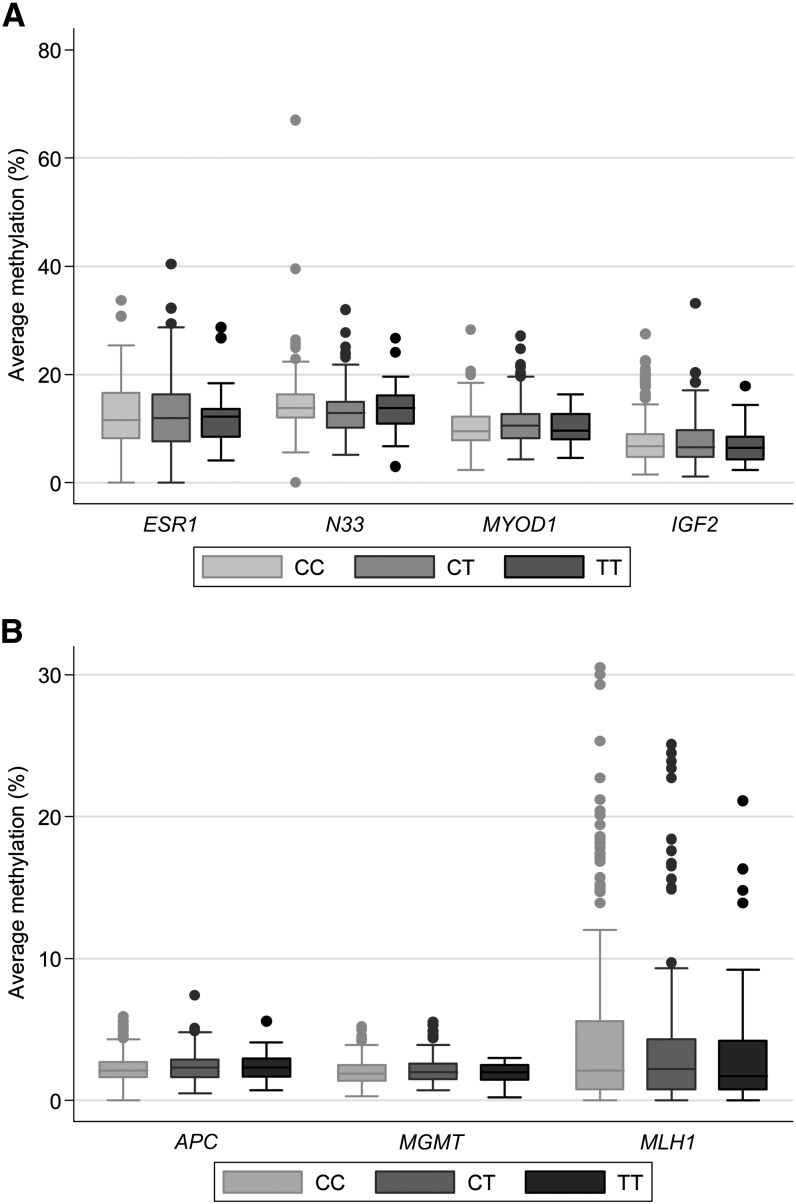
Gene-specific DNA methylation by *MTHFR* 677C>T genotype. Boxes represent medians and IQRs; whiskers are the expected range and outliers. A: *ESR1*, *N33*, *MYOD1*, and *IGF2* methylation. B: *APC*, *MGMT*, and *MLH1* methylation (CC, *n* = 185; CT, *n* = 119; TT, *n* = 32). Kruskal-Wallis tests were used for comparison between genotypes (*ESR1,* *P* = 0.92; *N33*, *P* = 0.12; *MYOD1,* *P* = 0.24; *IGF2,* *P* = 0.77; APC, *P* = 0.45; *MGMT,* *P* = 0.68; *MLH1,* *P* = 0.89).

**FIGURE 3.**
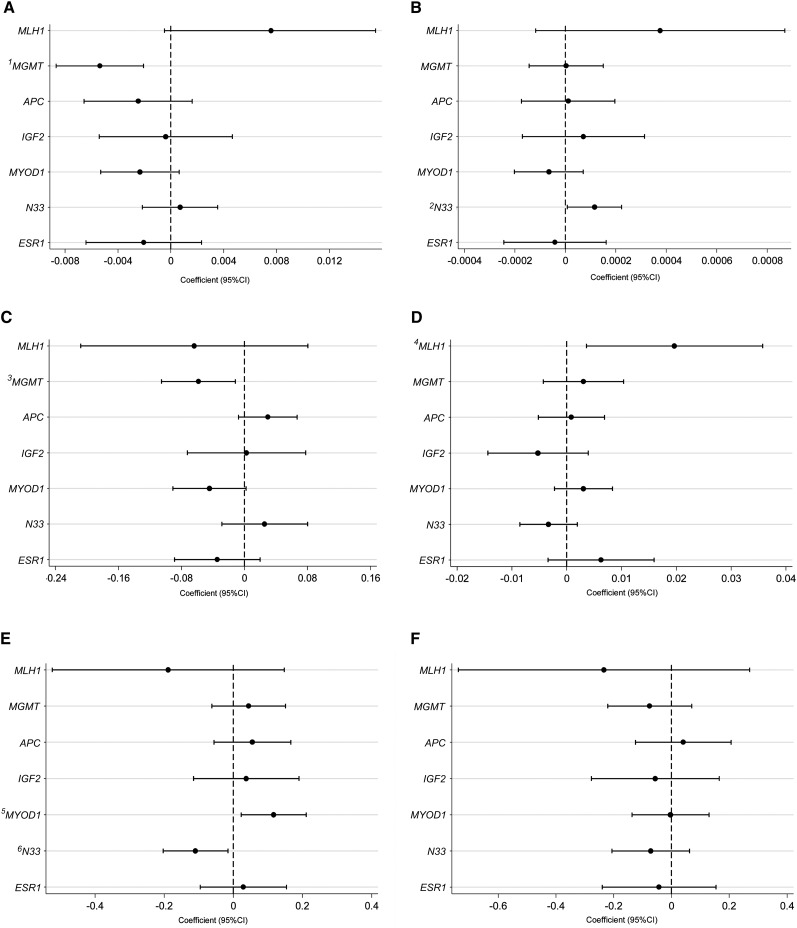
Association between gene-specific methylation in colonic mucosa, biomarkers of folate status, and *MTHFR* 677C>T genotype. Dots and horizontal lines represent the coefficients and 95% CIs, respectively, obtained from generalized linear models adjusted for age, sex (male or female), ethnicity (white or nonwhite), supplement use (user or nonuser), serum vitamin B-12, and *MTHFR* 677C>T genotype. The coefficients give the amount of change in the log odds of having high methylation in each gene investigated for each unit increase in the independent variables (with all other variables held constant) serum folate (nmol/L) (A), red blood cell folate (nmol/L) (B), tissue folate (nmol/g tissue) (C), and plasma homocysteine (μmol/L) (D) or the log odds of having high methylation in individuals carrying the *MTHFR* CT genotype (E) or TT genotype (F) compared with individuals carrying the CC genotype. The vertical dotted line (coefficient of 0) shows the line of no effect. Coefficients >0 indicate an increased odds of having high methylation (positive association), whereas coefficients <0 indicate a decreased odds of having high methylation (negative association), for each unit increase in biomarker concentrations, or for the CT or TT genotype compared with the CC genotype (reference). The threshold for significance with Bonferroni correction for 8 repeated tests is *P* = 0.006. ^1^*P* = 0.001 (significant after Bonferroni correction); ^2^*P* = 0.04; ^3^*P* = 0.01; ^4^*P* = 0.02; ^5^*P* = 0.02; ^6^*P* = 0.02.

Gene-specific methylation did not differ between CC, CT, and TT genotypes (**Figure 2**). The coefficients and 95% CIs for the association between gene-specific methylation and biomarkers of folate status and *MTHFR* 677C>T genotype, assessed by using generalized linear models (*see* Supplementary Tables 1 and 2 under “Supplemental data” in the online issue), are shown in **Figure 3** (A-F). The TT genotype did not influence methylation in any of the genes investigated (Figure 3F). However, the CT genotype was associated with lower *N33* methylation (*P* = 0.02) and higher *MYOD1* methylation (*P* = 0.02) (Figure 3E), although these relations were not significant after the Bonferroni correction for multiple comparisons (which raised the threshold for significance to *P* = 0.006).

A positive association was found between red blood cell folate and *N33* methylation (*P* = 0.04) (Figure 3B), a negative association between serum (Figure 3A) and tissue (Figure 3C) folate and *MGMT* methylation (*P* = 0.001 and *P* = 0.01, respectively), and a positive association between plasma homocysteine and *MLH1* methylation (*P* = 0.02) (Figure 3D). Of these associations, only that between *MGMT* methylation and serum folate (*P* = 0.001) remained significant after Bonferroni correction. No other significant associations were noted.

## DISCUSSION

In the current study, we investigated the association between *MTHFR* 677C>T genotype and biomarkers of folate status and genomic and gene-specific methylation in the colon in 336 subjects without colorectal neoplasia. This population was replete with regards to folate and vitamin B-12 status. The distribution of the *MTHFR* T allele (35% CT and 10% TT) in our population was in Hardy-Weinberg equilibrium (χ^2^ = 3.80).

The *MTHFR* TT genotype was not associated with either genomic- or gene-specific methylation in the colon. This result confirms our previous finding of no association between the *MTHFR* TT genotype and either genomic- (15) or gene-specific (18) DNA methylation in the colon in a smaller sample of subjects without neoplasia (68 and 73 subjects, respectively). Although there was a suggestion of a relation between the CT genotype and *N33* and *MYOD1* methylation, the relevance of this is not clear, particularly because this relation was not consistent and not significant after Bonferroni correction for multiple comparisons. Only 3 studies have investigated the association between *MTHFR* 677C>T genotype and DNA methylation, but in individuals with adenoma and cancer. Figueiredo et al (16) and Wallace et al (17) reported no association between *MTHFR* 677C>T genotype and long interspersed nuclear element-1 (*LINE-1*) methylation (a measure of genomic DNA methylation) and *ESR1* and *SFRP1* methylation, respectively, in the colonic mucosa of 388 subjects with previously resected colorectal adenoma. Iacopetta et al (34) measured *LINE-1* methylation in the normal mucosa of 178 individuals with colorectal cancer and showed that individuals with the mutant alleles for both *MTHFR* 677C>T and A1298C (ie, the low-activity genotype 677TT/1298CC) had lower LINE-1 methylation than did those with a high-activity genotype (677CC/1298AA) (*P* = 0.03). It is possible that having 2 mutations exerts a stronger effect on DNA methylation than does either mutation in isolation or that the relation between *MTHFR* 677C>T genotype and DNA methylation differs in subjects with and without cancer.

Our finding of no association between *MTHFR* TT genotype and DNA methylation is confirmed by other studies in healthy subjects that have measured DNA methylation in lymphocyte DNA (35–39). As with our study population, subjects in these studies were folate replete. In subjects with poor folate status, the TT genotype does influence genomic DNA methylation in lymphocytes (24, 40–42), and TT individuals have lower levels of genomic DNA methylation (24, 42) or show greater increases in genomic DNA methylation in response to folate repletion (40, 41). It is possible that an association between *MTHFR* 677C>T genotype and DNA methylation in the colon is only evident in individuals with low folate status. Friso et al (24) investigated the interaction between folate status and *MTHFR* 677C>T genotype in determining genomic DNA methylation in 187CC and 105TT subjects. In their study, the difference in genomic DNA methylation in lymphocytes between CC and TT individuals was observed only when serum folate was below the median (<12 nmol/L). In our study the median serum folate concentration was 25 nmol/L, which is more than double that reported by Friso et al (24). When we stratified our population using the cutoff value used by Friso et al, we observed a 26% decrease in genomic DNA methylation between CC and TT subjects in those with serum folate values <12 nmol/L, although the number of subjects was too small (12 CC and 2 TT) to allow a statistical comparison.

Another possible reason for the lack of association between the *MTHFR* TT genotype and DNA methylation in our study was the lack of differences in serum and red blood cell folate between genotypes. Although plasma homocysteine was 13% higher in TT individuals than in CC individuals, this difference was not statistically significant. Generally, although population studies have shown that plasma homocysteine is higher in TT individuals (43), this effect is modified by folate status—a high folate status is associated with a reduced effect of *MTHFR* 677C>T on homocysteine concentrations (44–46). In our multiple linear regression analysis, age, sex, and serum folate had stronger effects on plasma homocysteine concentrations (*P* ≤ 0.01) than did *MTHFR* 677C>T genotype (*P* > 0.05).

The current study showed no association between folate biomarkers and genomic DNA methylation in colonic mucosa (Table 3) after adjustment for various factors. In the unadjusted model, red blood cell folate was negatively associated with genomic DNA methylation (ie, DNA becomes hypomethylated as red blood cell folate increases). This is contrary to our previous report in which we showed a weak positive association between serum and red blood cell folate and genomic DNA methylation (*P* = 0.07 and *P* = 0.08, respectively) in subjects without neoplasia (15). In that study, we used the methyl acceptance assay to measure DNA methylation, and, although studies using the methyl acceptance assay have shown associations between folate status and DNA methylation (19, 20, 47), this has not been replicated in studies using other methylation measurements (16, 23, 48).

We measured gene-specific methylation in 7 genes (*ESR1*, *MYOD1*, *IGF2*, *N33*, *APC*, *MLH1*, and *MGMT*). The first 4 genes (*ESR1*, *MYOD1*, *IGF2*, and *N33*) had a wide range of methylation values (1–67%), and all except *IGF2* were strongly associated with age (*P* < 0.001). The latter 3 genes (*APC*, *MLH1*, and *MGMT*) were not associated with age and had a narrow range of methylation values (0–7%), except for *MLH1*, for which a proportion of subjects (19%) had methylation values >10%. These findings are similar to what has been previously reported in the literature for these genes (28, 49, 50).

We found no significant associations between markers of folate status and methylation of *ESR1*, *MYOD1, IGF2*, and *APC* in the colon. For *N33*, *MGMT*, and *MLH1*, some associations were noted, although these were in opposite directions (increased methylation with higher red blood cell folate for *N33* and decreased methylation with higher serum and colonic folate for *MGMT*). The importance of these findings is not certain, given that these relations were not consistent, either between genes or for biomarkers within the same gene and all but one of these associations (*MGMT* methylation and serum folate) were not significant after correction for multiple comparisons. In our previous study, we found no relation between markers of folate status and *ESR1* and *MLH1* methylation in 73 subjects without colorectal neoplasia (15). Wallace et al (17) reported that higher red blood cell folate concentrations were associated with higher levels of *ESR1* (*P* = 0.03) and *SFRP1* (*P* = 0.01) methylation in normal colonic mucosa of subjects with adenoma, which suggests a negative effect of high folate status on gene-specific methylation. However, this study included subjects with previously resected adenomas who participated in a randomized controlled trial of folic acid supplementation (1 mg/d for 3 y) for the prevention of further adenomas. Individuals with adenoma may have different gene-specific methylation patterns in their normal mucosa than individuals who are neoplasia free [as has been shown previously (12, 15, 26)] and so may respond differently to increasing folate status, particularly when folic acid is administered in pharmacologic doses.

Our study had several strengths. We used a population without neoplasia, which avoided the potential confounding effects that neoplasia may have on DNA methylation. We also used quantitative assays with high sensitivity to assess genomic and gene-specific DNA methylation. We measured both systemic and tissue folate to allow a comprehensive assessment of the relation between folate status and DNA methylation. Colonic tissue folate was strongly associated with serum and red blood cell folate, so it appears that systemic markers of folate status are good surrogates of folate status in the colon. The limitations of our study include the fact that our population may not be representative of general population without neoplasia, because they presented with symptoms (eg, bleeding, change in bowel habit, pain, and bloating) that prompted referral for colonoscopy. These individuals may have different dietary habits and other factors that may have influenced DNA methylation in the colon. Another limitation is that none of the methylation assays were performed in duplicate because of the limited availability of colonic tissue biopsies. This may have influenced our findings in view of the variability in the methylation assays (7–10%). Also, we used rectal biopsies, but there is evidence that methylation levels differ between the left and right side of the colon (17).

In conclusion, the findings of the current study suggest that *MTHFR* 677C>T genotype and biomarkers of folate status do not influence DNA methylation in this population. The suggestion that a higher folate status is associated with lower *MGMT* methylation needs to be interpreted with caution given the inconsistencies across the folate measures and between different genes. Future studies need to examine these relations in populations with low folate status and examine the influence of other mutations in folate pathway genes on DNA methylation in the colon.

## Supplementary Material

Supplemental data

## Footnotes

5 Abbreviations used: *APC*, adenomatous polyposis coli; *ESR1,* estrogen receptor 1; *IGF2,* insulin-like growth factor II; *LINE-*1, long interspersed nuclear element-1; *MGMT*, O^6^-methylguanine-DNA methyltransferase; *MLH1*, mut-L homolog 1; *MTHFR*, methylenetetrahydrofolate reductase; *MYOD1*, myoblast determination protein 1; *N33,* tumor suppressor candidate 33; PCR, polymerase chain reaction; QC, quality control; *SFRP1*, secreted frizzled-related protein 1.
