# Supplementary material for: The association between MTHFR 677C>T genotype and folate status and genomic and gene-specific DNA methylation in the colon of individuals without colorectal neoplasia1
Source: Am J Clin Nutr. 2013 Oct 9;98(6):1564–74. doi: 10.3945/ajcn.113.061432 (PMC3831541; doi:10.3945/ajcn.113.061432)
Supplement: Supplemental data [file supp_98_6_1564__index.html]

Supplemental data 

# The association between *MTHFR* 677C>T genotype and folate status and genomic and gene-specific DNA methylation in the colon of individuals without colorectal neoplasia

## Supplemental data

**Files in this Data Supplement:**

- Supplemental data - Table 1
- Supplemental data - Table 2
