# Supplementary material for: The association between MTHFR 677C>T genotype and folate status and genomic and gene-specific DNA methylation in the colon of individuals without colorectal neoplasia1
Source: Am J Clin Nutr. 2013 Oct 9;98(6):1564–74. doi: 10.3945/ajcn.113.061432 (PMC3831541; doi:10.3945/ajcn.113.061432)
Supplement: Supplemental data [file 113.061432_ajcn061432SupplementaryData1.doc]

**Supplementary Table 1**. Association between *ESR1*, *N33*, *MYOD* and *IGF2* methylation, *MTHFR* C677T genotype and biomarkers of folate status

|  | *ESR1* |  | *N33* |  | *MYOD1* |  | *IGF2* |  |
| --- | --- | --- | --- | --- | --- | --- | --- | --- |
|  | Coefficient (95% CI) | P | Coefficient (95% CI) | P | Coefficient (95% CI) | P | Coefficient (95% CI) | P |
| *MTHFR* C677T^1^ |  |  |  |  |  |  |  |  |
| CC (reference) |  |  |  |  |  |  |  |  |
| CT | 0.029 (-0.096, 0.154) | 0.65 | -0.109 (-0.203, -0.016) | 0.02 | 0.117 (0.023, 0.211) | 0.02 | 0.038 (-0.115, 0.190) | 0.63 |
| TT | -0.043 (-0.240, 0.154) | 0.67 | -0.071 (-0.206, 0.062) | 0.30 | -0.003 (-0.137, 0.130) | 0.96 | -0.056 (-0.278, 0.165) | 0.62 |
| Serum folate (nmol/l) | -0.002 (-0.006, 0.002) | 0.36 | 0.001 (-0.002, 0.004) | 0.63 | -0.002 (-0.005, 0.001) | 0.12 | -0.000 (-0.005 0.005) | 0.88 |
| Red cell folate (nmol/l) | -0.000 (v -0.0001, 0.0002) | 0.69 | 0.0001 (0.000, 0.0002) | 0.04 | -0.0001 (-0.0002, 0.0001) | 0.35 | 0.0000 (-0.0001, 0.0003) | 0.56 |
| Colonic tissue folate (nmol/g tissue) | -0.034 (-0.089, 0.197) | 0.21 | 0.026 (-0.029, 0.080) | 0.36 | -0.044 (-0.091, 0.002) | 0.06 | 0.003 (-0.072, 0.078) | 0.94 |
| Plasma homocysteine (µmol/l) | 0.006 (-0.003, 0.016) | 0.20 | -0.003 (-0.009, 0.0019) | 0.21 | 0.003 (-0.002, 0.008) | 0.26 | -0.005 (-0.014, 0.004) | 0.26 |

Generalized linear model with a logit link (the natural log of the odds, i.e. log$\left\{\frac{p}{1- p}\right\}= \beta_{0}+ \beta1X_{1}+ \beta2X_{2}\ldots\beta kX_{k}$). All models are adjusted for age, gender (male or female), ethnicity (white or non-white), supplement use (user or non-user), serum vitamin B_12_ and *MTHFR* C677T genotype.

For the continuous predictor variables (biomarkers of folate status), the coefficient gives the amount of change in the log odds of having high methylation for each unit increase in the biomarker (i.e. a positive coefficient suggests an increase in the odds of having high methylation with increasing biomarker concentration). For the categorical predictor variable (*MTHFR* C677T), the coefficient gives the log odds of having high methylation in the CT or TT genotype compared with the CC genotype (reference group) (i.e. a negative coefficient suggests lower odds of having high methylation in individuals carrying the TT genotype compared with individuals carrying the CC genotype).

Threshold for significance using Bonferroni correction for 8 repeated tests is P= 0.006.

^1^Adjusted for age, gender (male or female), ethnicity (white or non-white), supplement use (user or non-user) and serum vitamin B_12_.
